# Protein-losing enteropathy and joint contractures caused by a novel homozygous ANTXR2 mutation

**DOI:** 10.2147/AGG.S159077

**Published:** 2018-06-27

**Authors:** Edith Schussler, Rita V Linkner, Jacob Levitt, Lakshmi Mehta, John A Martignetti, Kimihiko Oishi

**Affiliations:** 1 Department of Pediatrics, Icahn School of Medicine at Mount Sinai, New York, NY, USA;; 2 Department of Dermatology, Icahn School of Medicine at Mount Sinai, New York, NY, USA;; 3 Department of Genetics and Genomic Sciences, Icahn School of Medicine at Mount Sinai, New York, NY, USA

**Keywords:** infantile systemic hyalinosis, hyaline fibromatosis syndrome, juvenile hyaline fibromatosis, *ANTXR2*, enteropathy

## Abstract

Infantile systemic hyalinosis (ISH) is a rare autosomal recessive disorder and an allelic form of hyaline fibromatosis syndrome that is caused by mutations in the *ANTRX2* gene encoding the transmembrane anthrax toxin receptor 2. Its main features include characteristic skin lesions, joint contractures, persistent diarrhea, and failure to thrive due to accumulation of hyaline material in multiple organs. The resulting severe malnutrition can cause death in early infancy. Because of its rarity and high fatality rate, timely diagnosis is difficult and ISH may be underdiagnosed. In this report, we describe a 10-month-old male with severe protein-losing enteropathy, skin lesions, and painful joint contractures, diagnosed with ISH based on skin his-topathology and identification of a novel homozygous *ANTRX2* mutation, c.1127_1128delTG (p.V376Gfs*14). While its clinical outcome is poor without curative treatment, establishing a diagnosis of ISH starting from clinical suspicion to molecular analysis is important for appropriate medical management and for risk and carrier assessment of family members.

## Introduction

Infantile systemic hyalinosis (ISH) (MIM #228600) is a rare autosomal recessive disorder characterized by painful joint contractures, skin hyperpigmentation over bony prominences, thickening of the skin with pearly papules, osteoporosis, bone fractures, persistent diarrhea, and failure to thrive.^1,2^ ISH is an allelic form of hyaline fibromatosis syndrome^3^ caused by mutations in the *ANTRX2* (also known as *CMG2)* gene (MIM #608041), which encodes the transmembrane anthrax toxin receptor 2/ capillary morphogenesis protein-2.^4^ ISH generally results in life-threatening malnutrition that leads to early death in infancy, unlike its milder allelic condition, juvenile hyaline fibromatosis (JHF).^2,5,6^ While the genetic basis is now known, the molecular pathogenesis of hyaline fibromatosus syndrome is unclear. It is hypothesized that disruption of this membrane protein causes accumulation of hyaline materials and tissue damage/malfunction in multiple organs.^7^ Owing leading to identification of a novel diagnostic homozygous mutation, c.1127_1128delTG (p.V376Gfs*14), in *ANTRX2.*

## Case report

**Figure 1:**
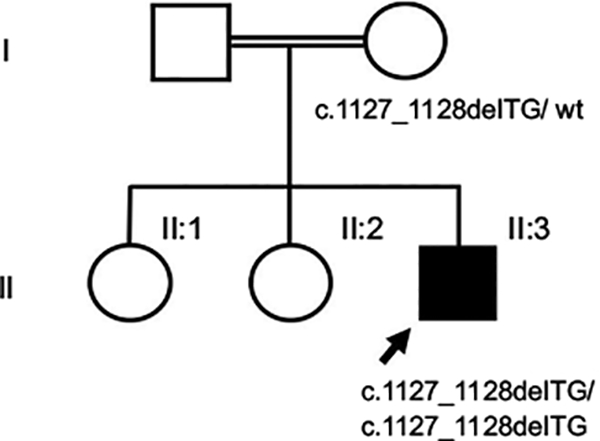
Pedigree and genotypes. **Notes:** The affected patient is indicated by the arrow and filled square. The parents are consanguineous, as shown with a double horizontal line. The patient was homozygous, and the mother was heterozygous for the novel *ANTRX2* mutation, c. I 127I 128delTG (p.V376Gfs*l4).

The patient was an Asian 10-month-old male (individual II-3 in Figure 1) who was bom full term with a birth weight of 3.75 kg (66 percentile). His parents were half-first cousins and had 2 healthy daughters (Figure 1). The proband had mild contractures in major joints at birth but was otherwise noted to be healthy. At 5 months of age, his joint contractures had progressively worsened and were associated with pain in his wrists, elbows, shoulders, hips, knees, and ankles. Concurrently, he developed dark brown/black spots on his knuckles, ankles, back, and neck. Initially, arthrogryposis was suspected, and so he received physical therapy, which resulted in a femur fracture. At that time, he also developed persistent protein-losing enteropathy with significant weight loss. He came to the USA for additional medical care at the age of 10 months. According to the family, there were no other family members who had similar symptoms as the patient.

**Figure 2:**
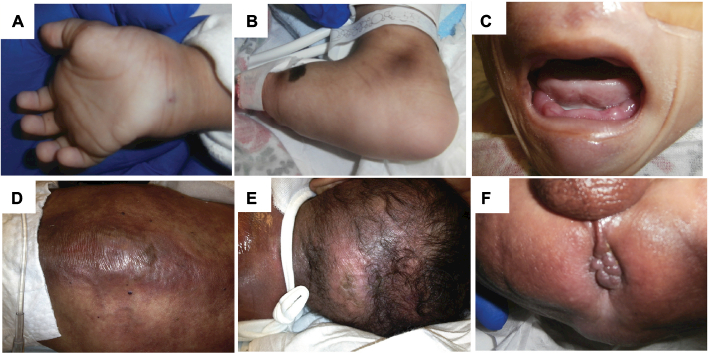
Clinical findings of patient and skin biopsy. **Notes:** Contractures of the wrists and ankles **(A** and **B).** There were generalized sclerodermatous changes of the skin also appreciated in the extremities, most prominently in the left lower extremity **(B).** Gingival hyperplasia **(C).** Skin findings on the back **(D),** posterior scalp **(E),** and perineum perianal area **(F).**

On presentation, the most striking features were the severe malnutrition (5 kg; <3 percentile) and constant irritability. On physical examination, significant joint contractures of the wrists, knees, hips, and ankles were noted (Figure 2A and B). Oral mucosa demonstrated gingival hyperplasia (Figure 2C). There were generalized sclerodermatous changes of the skin, most prominently in the left lower extremity (Figure 2B ). Skin was significant for pearly, erythematous papules and indurated plaques located symmetrically on the back (Figure 2C and D). Similar indurated plaques that were more erythematous than violaceous were also seen on the posterior scalp (Figure 2E). The perianal area revealed multiple coalescent skin-colored, indurated papules involving the perineum (Figure 2F ). Due to long-standing malnutrition, the child had multiple electrolyte abnormalities including a nonanion gap acidosis, hyponatremia, hyperkalemia, and hypoalbuminemia.

**Figure 3:**
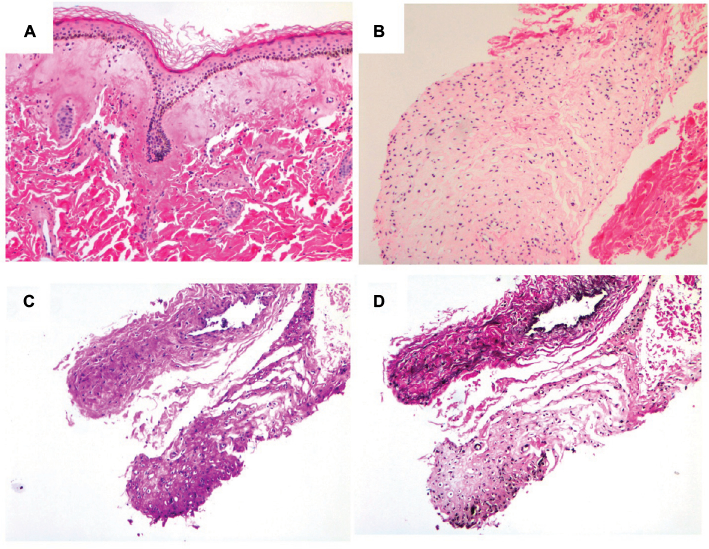
Skin pathology. **Notes:** Skin biopsy from the infant’s back with hematoxylin and eosin stain (**A** and **B**). Some of the cells in the hyaline material appeared to lie in lacunae, giving it a chondroidlike appearance **(B).** Periodic acid-Schiff staining (C), and Verhoeff’s Van Gieson stain **(D).** Original magnification x100.

A biopsy was obtained from one of the violaceous, indurated plaques on the infant’s back, and histopathologic analysis revealed an amorphous eosinophilic, hyaline material with spindle cells deposited in the superficial and deep dermis consistent with ISH (Figure 3A and B). Some of the cells in the hyaline material appeared to lie in lacunae, giving it a chondroid-like appearance (Figure 3B). The deposits were Periodic acid-Schiff staining positive (Figure 3C), diastase resistant, and did not stain with Alcian blue. No elastic fibers were identified on a Verhoeff’s Van Gieson stain (Figure 3D). These findings were consistent with ISH.

**Figure 4:**
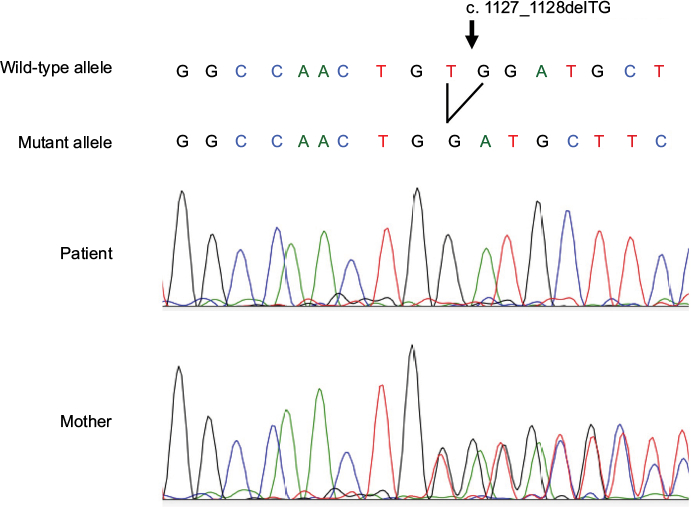
Sanger sequencing of *ANTRX2* gene. **Notes:** Sanger sequencing of the patient’s exon 14 of *ANTRX2* gene revealed a homozygous 2-bp deletion at nucleotide 1 128, c. 1127_128delTG (p.V376Gfs*l4). This frameshift mutation results in a presumed truncated protein. Sequencing of mother’s genomic DNA was heterozygous for the mutation.

To confirm the diagnosis, we sequenced all 17 coding exons and intron/exon boundaries, of the *ANTRX2* gene using genomic DNA isolated from the patient and his mother. A homozygous 2-bp TG deletion in exon 14, which predicted a frameshift mutation, c. 1127_1128delTG (p.V376Gfs* 14), was identified in the patient. Consistent with her presumed carrier status, the mother was heterozygous for the *ANTRX2* mutation (Figures 1 and 4). The father’s sample was not available for analysis. Written informed consent for publication of the patient’s clinical information, including photographs, was obtained from the patient’s parents.

## Discussion

The approach to diagnosing rare disorders can be particularly difficult. Identifying certain characteristic features can help navigate toward the selection of the appropriate targeted molecular tests. For our case, the combination of skin lesions,joint contractures, gingival hyperplasia, persistent diarrhea, and failure to thrive led to sequencing of the *ANTRX2* gene and identification of a homozygous frameshift mutation in exon 14. The never previously reported c.1127_1128delTG (p.V376Gfs*14) mutation is believed to be deleterious, and the etiology of ISH due to its predicted truncation of the protein. Since the original identification of *ANTRX2* as the disease- causing gene for both ISH and the allelic disorder JHF,^4^ at least 37 different ISH/JHF-causing mutations in 67 patients and 49 families have been identified.^6,8–15^ Nearly all of the reported mutations are exonic and encompass missense, nonsense, and frameshift mutations. While disease-causing mutations have been identified in exons 1 to 15, a hotspot in exon 13 accounts for about 60% of ISH/JHF cases.^3,6–9^ To date, no significant phenotype-genotype correlation has been identified; however, most ISH affecteds have nonsense or frameshift mutations in the von Willebrand A domain, whereas JHF affecteds have missense mutations in other domains.^6–8,11^

The number of reported ISH/JHF cases remains limited, and its disease prevalence is unclear.^6^ As would be expected for a rare disorder, cases of ISH/JFH are probably underreported. Of interest, there have been an increasing number of cases in the literature with compound heterozygous *ANTRX2* mutations, and these account for nearly 40% of patients.^6,9^ Particularly notable is that a number of these mutations cluster in 1 hotspot, and these include c.1074delT, c.1073_1074insC, and c.1073_1074insCC.^6^ This suggests that the variant allele frequencies in this coding region may be more common than previously appreciated. According to data from the Exome Aggregation Consortium, a frameshift variant in the same exon, c.1070C>T (p.A357Pfs*52), was observed with an allele frequency of 0.0057.^16^ This variant has not yet been reported in ISH/JHF, but the predicted frame- shift suggests loss of protein function. Thus, the prevalence of carriers of deleterious hotspot mutations is most likely higher than appreciated. Given the high frequency of compound heterozygosity within one of the hotspot mutations and the early mortality associated with ISH, we hypothesize that ISH may be more frequently encountered in practice without a diagnosis being established.

Interestingly, the novel frameshift truncating mutation we identified, p.V376Gfs*14, is predicted to reside in the protein’s cytoplasmic domain. While JHS-causing mutations have been more commonly found in this domain, our patient’s clinical symptoms and disease course are consistent with ISH.^6^ Thus, our patient’s disease-causing mutation, along with 2 previously reported truncation mutations for ISH in the same cytoplasmic domain,^11,17^ highlights the functional importance of the C-terminal structure of the protein. Intriguingly, while the role of the ANTRX2 protein as an anthrax toxin receptor is known, the molecular derangements resulting in the unique symptoms of ISH/JHF, and possible phenotype/genotype correlation, are poorly understood.^18^ The protein’s von Willebrand A domain, which binds to both lamin and collagen IV, suggests its role in basement membrane matrix assembly and endothelial cell morphogenesis, resulting in tissue damage and/or multiple organ malfunction.^4,7^ Despite severe musculoskeletal involvement, cognitive function is most likely spared because of the lack of ANTRX2 protein expression in the brain.^2,19^

In general, precise molecular diagnosis is imperative not only for medical management of the patient but also for risk and carrier assessment of family members. Our patient’s condition at the time of presentation to our institution and diagnosis was refractory to nutritional support and electrolyte correction, and he died at 11 months of age. It is unknown if earlier attempts at parenteral nutrition would have changed this outcome. A recent study suggested that proteasome inhibitors might represent therapeutic candidates depending on the severity of the *ANTRX2* mutation.^9,20^ Ultimately, if clinical suspicion leads to earlier molecular diagnosis of ISH, this would allow for more meaningful assessment of candidate therapeutics in treatment and support of this disease.
